# Mitochondrial Ca^2+^ in Cancer Growth and Metabolism

**DOI:** 10.1002/jcp.70093

**Published:** 2025-09-23

**Authors:** Jillian S. Weissenrieder, J. Kevin Foskett

**Affiliations:** ^1^ Department of Physiology University of Pennsylvania Perelman School of Medicine Philadelphia Pennsylvania USA; ^2^ Department of Cell and Developmental Biology University of Pennsylvania Perelman School of Medicine Philadelphia Pennsylvania USA

**Keywords:** Ca^2+^ signaling, cancer, metabolism, mitochondria

## Abstract

Cancer is a leading cause of death in developed countries, despite many breakthroughs in targeted small molecule and immunotherapeutic interventions. A deeper understanding of the characteristics and processes that underlie malignancy will enable us to develop more effective therapeutic options to improve patient outcomes. One particular area of interest is in cancer cell metabolism. Even as early as the 1920s, Otto Warburg recognized dysregulated metabolism in cancerous cells. Altered metabolism may provide targetable nutrient dependencies for further clinical development, either by nutrient restriction or pathway inhibition. More recently, researchers have observed an increasingly strong linkage between altered mitochondrial Ca^2+^ homeostasis and tumor cell metabolism, with strong implications for therapeutic targeting. In this review, we summarize the literature surrounding mitochondrial Ca^2+^ homeostasis, metabolism, and cancer, as well as providing a discussion of the potential for mitochondrial Ca^2+^ modulation as an anticancer therapeutic modality.

## 1 Introduction

Cancer, a disorder characterized by uncontrolled division of cells, is a leading cause of death globally, with > 50,000 deaths in 2024 in the United States of America alone (SEER 2024). Traditionally, patients have been treated with broad‐based chemotherapeutics, such as platinum‐based compounds that target cell division (Anand et al. 2023). These agents have unpleasant side effects since they also target dividing normal cells (Anand et al. 2023). More recently, targeted small molecule therapies, such as receptor tyrosine kinase (RTK) inhibitors, and immunotherapeutic agents have been developed and entered widespread use (Anand et al. 2023). While such targeted therapies have increased life expectancy and quality of life in cancer patients, many still suffer from poor outcomes due to treatment resistance, both inherent and induced in response to therapy (Sabnis and Bivona 2019; Boumahdi and de Sauvage 2020). As such, novel therapies are still needed for many patients.

It has been long appreciated that growth and metabolism differ between normal cells and cancer cells. In the 1920s, Otto Warburg noticed that tumors consumed high amounts of glucose and produced high levels of lactate, regardless of O_2_ availability, a phenomenon now called the “Warburg effect” (Hardie 2022; Warburg 1956a, 1956b). Warburg hypothesized that this “aerobic glycolysis” was caused by a respiratory deficit. It was previously believed that mitochondrial metabolism, including the tricarboxylic acid (TCA) cycle and the electron transport chain (ETC), were defective in cancer cells, (Hardie 2022; Warburg 1956a, 1956b; Bose et al. 2021; Vaupel and Multhoff 2021; Ward and Thompson 2012). However, it is now appreciated that these pathways remain functional in cancer cells and that ATP supplied by oxidative phosphorylation (OXPHOS) may be as important as that supplied by aerobic glycolysis in many cancers (Hardie 2022; Bose et al. 2021; Vaupel and Multhoff 2021; Ward and Thompson 2012). Furthermore, recent studies have clarified that the TCA cycle is critical in cancer cells for the synthesis of biosynthetic intermediates necessary for the production of building blocks to support cell growth, including nucleotides and proteins, as well as for maintaining redox homeostasis, rather than or in addition to serving as a source of reducing equivalents for the ETC (Arnold and Finley 2023; Georgakopoulos‐Soares et al. 2020; Martínez‐Reyes and Chandel 2020; Zong et al. 2016). Metabolites produced in the TCA cycle can promote cancer development through alterations in reactive oxygen species (ROS) production, transcription factor activation, and support of the biosynthesis of amino acids and nucleic acids necessary for cell growth and development (Wu 2024). Recent studies have elucidated the regulation of mitochondrial function and metabolic processes by Ca^2+^ signaling. In this review, we will discuss the relationship between mitochondrial Ca^2+^ homeostasis and metabolic functions in cancer, and the potential for targeting this axis in cancer.

## 2 Mitochondria as an Organelle

Mitochondria are organelles within eukaryotic cells that consist of an outer membrane (OMM) and an inner membrane (IMM) that surrounds a matrix. The IMM is highly involuted, forming cristae that contain respiratory chain complexes (Al Amir Dache and Thierry 2023; Annesley and Fisher 2019). Cristae structures can be dynamically regulated in response to energy demands (Al Amir Dache and Thierry 2023; Annesley and Fisher 2019; Protasoni and Zeviani 2021). Mitochondria possess their own subset of DNA (mtDNA) that exists as a circular chromosome and codes for polypeptides required for OXPHOS, as well as for transfer and ribosomal RNAs (Protasoni and Zeviani 2021; Gustafsson et al. 2016). Because of the existence of mtDNA, the dual‐membrane structure, and phylogenetic analysis, mitochondria are thought to be descended from endosymbiotic proteobacteria (Martin et al. 2015). Mitochondria are typically passed from mother to child, with no input from paternal mtDNA (Giles et al. 1980). Thus, mitochondrial disorders resultant from mutations in mtDNA are passed down through the maternal line (Annesley and Fisher 2019; Giles et al. 1980). Such disorders, as well as mutations in nuclear‐encoded mitochondrial genes, may alter the apparent morphology and function of mitochondria (Protasoni and Zeviani 2021). The mitochondrial network is in dynamic flux. Mitochondria undergo fission and fusion to regulate function and to respond to stress (Protasoni and Zeviani 2021). In addition, damaged mitochondria undergo mitophagy, a specialized form of autophagy in which mitochondria are “self‐eaten” by the cell (Kim et al. 2007; Lemasters 2005). Mitochondrial biogenesis also contributes to the highly dynamic nature of these organelles (Quintana‐Cabrera and Scorrano 2023). Mitochondria may be large, small, interconnected, swollen and lozenge‐like, or narrow and long in shape, with many or few cristae (Quintana‐Cabrera and Scorrano 2023). Such structural differences are tied to alterations in metabolic function and nutrient availability (Giacomello et al. 2020). Highly networked mitochondria may support ATP generation when cells are in more hypoxic environments, while fragmented mitochondria are associated with both stress responses and nutrient‐replete conditions (Giacomello et al. 2020; Chen et al. 2023a). Higher numbers of cristae increase mitochondrial efficiency (Giacomello et al. 2020; Chen et al. 2023a). Notably, these changes are linked to not only changes in nutrient availability, but are also affected by Ca^2+^ signals. For instance, elevated cytoplasmic Ca^2+^ concentrations ([Ca^2+^]_i_) associated with cell signaling may promote mitochondrial fission (Hom et al. 2007). It is thought that such dynamism is critical in cancer cells, as it allows them to rapidly adapt to changing conditions and stress to promote survival (Zong et al. 2016; Wallace 2012).

## 3 Mitochondrial Metabolism

Mitochondria are often thought of primarily as intracellular power sources due to their key role in ATP synthesis (Alberts 2008; Lehninger et al. 2013). Mitochondria also contribute significantly to the production of biosynthetic intermediates required for the synthesis of proteins, lipids, and nucleotides (Birsoy et al. 2015; Sullivan et al. 2015; DeBerardinis and Chandel 2016; Calhoun et al. 2022; Castro et al. 2012; Hlavaty et al. 2024; Xu et al. 2024a) critical for cell growth and division. The production of these intermediates may be of more importance in cancer cells than in normal cells, since cancer cells grow and divide more rapidly, necessitating the synthesis of more membranes, RNA, DNA, and proteins (DeBerardinis and Chandel 2016). Breakdown of glucose, fatty acids, and glutamine in mitochondria support biochemical intermediate fluxes through the TCA cycle, which represents the main pathway by which cells process acetyl‐CoA derived from carbohydrate and lipid metabolism to provide electron equivalents in the form of NADH and FADH_2_ to the ETC (Arnold and Finley 2023; Martínez‐Reyes and Chandel 2020), where they are ultimately consumed to manufacture ATP, generating ROS in the process. The TCA cycle is able to regenerate itself, and intermediates can also be replenished as needed through the process known as anapleurosis (Kaadige et al. 2009; Cheng et al. 2011; Christen et al. 2016; Kiesel et al. 2021). All these processes are, in one way or another, regulated by Ca^2+^ signaling.

## 4 Ca^2+^ Signal Transduction to Mitochondria

Ca^2+^ serves as a critical second messenger in cells, regulating a variety of enzymes, transcription factors, and ion channels necessary for cellular growth, metabolism, and survival (Garbincius and Elrod 2022). [Ca^2+^]_i_ is highly regulated in a cell type, cell state, and cell compartment ‐regulated manner, with concentrations generally maintained within a set range. When [Ca^2+^]_i_ deviates from these acceptable ranges, it may restrict cellular function, division, and/or survival (D'Angelo et al. 2023). Basal [Ca^2+^]_i_ is maintained at ~100 nM while extracellular [Ca^2+^] is typically a 1–4 mM (Raffaello et al. 2016). Cells maintain low [Ca^2+^]_i_ by active Ca^2+^ extrusion across the plasma membrane and by active sequestration into the sarco/endoplasmic reticulum (ER), the major intracellular Ca^2+^ storage organelle (Brini and Carafoli 2011; Niggli et al. 1982; Hao et al. 1994), processes that are energetically demanding. ER luminal free [Ca^2+^] is generally in the 100‐1000 μM range, with total Ca^2+^ content even higher as a consequence of significant sequestration by luminal Ca^2+^‐binding proteins (Garbincius and Elrod 2022). As discussed in more detail below, mitochondrial matrix [Ca^2+^] ([Ca^2+^]_m_) is also highly regulated, with resting [Ca^2+^]_m_ generally maintained ~100–500 nM. Importantly, [Ca^2+^]_i_ signals can be transmitted to mitochondria, raising [Ca^2+^]_m_ to higher levels, with important functional effects (Raffaello et al. 2016; Chalmers and Nicholls 2003; Wei et al. 2012).

The large electrochemical gradients for Ca^2+^ between the extracellular milieu and cytoplasm, and between the ER lumen and cytoplasm, enable the activation of Ca^2+^ permeabilities in these membranes to cause rapid changes in [Ca^2+^]_i_ as Ca^2+^ moves down these gradients. [Ca^2+^]_i_ signals can be activated by the opening of Ca^2+^‐permeable plasma membrane ion channels or through the release of Ca^2+^ from intracellular stores, predominately the ER, through Ca^2+^‐release ion channels (Raffaello et al. 2016). ER‐dependent Ca^2+^ signaling can be stimulated when RTKs and Gαq‐coupled G‐protein coupled receptors (GPCRs) in the plasma membrane are stimulated by extracellular signals (Raffaello et al. 2016; Evans et al. 1985). Resultant conformational changes in the receptors may couple to phospholipase C (PLC) activation (Harden et al. 2011). Conformational changes in PLC catalyze the hydrolysis of phosphatidylinositol‐4,5‐bisphosphate (PIP_2_) to diacylglycerol (DAG) and inositol‐1,4,5‐trisphosphate (IP_3_) (Harden et al. 2011). IP_3_ diffuses through the cytoplasm and binds to the IP_3_ receptor (IP_3_R), an ER‐resident Ca^2+^‐permeable ion channel, activating it to enable release of ER Ca^2+^ into the cytoplasm (Streb et al. 1983). Elevated [Ca^2+^]_i_ can be sensed by cytoplasmic effectors and be transferred to organelles including the nucleus, lysosomes and mitochondria. Receptor‐activated [Ca^2+^]_i_ signals can be highly localized or propagate throughout the cell, be transient or sustained, and vary temporally as oscillations or spikes. Sustained activation that results in a decrease in [Ca^2+^]_ER_ can result in activation of store‐operated Ca^2+^ entry (SOCE), in which ER‐localized STIM (stromal interaction molecule) Ca^2+^ sensors and plasma membrane ORAI (Ca^2+^ release‐activated calcium channel) Ca^2+^ channels cooperate to sustain the [Ca^2+^]_i_ signal and to replenish ER stores upon termination of the stimulus to prime the system for future rounds of signaling (Putney 1986; Wang et al. 2009). For further information on the regulation of SOCE, see Lewis's 2020 review on the subject (Lewis 2020).

## 5 Ca^2+^ Fluxes at the Mitochondrial Membrane

**Figure 1:**
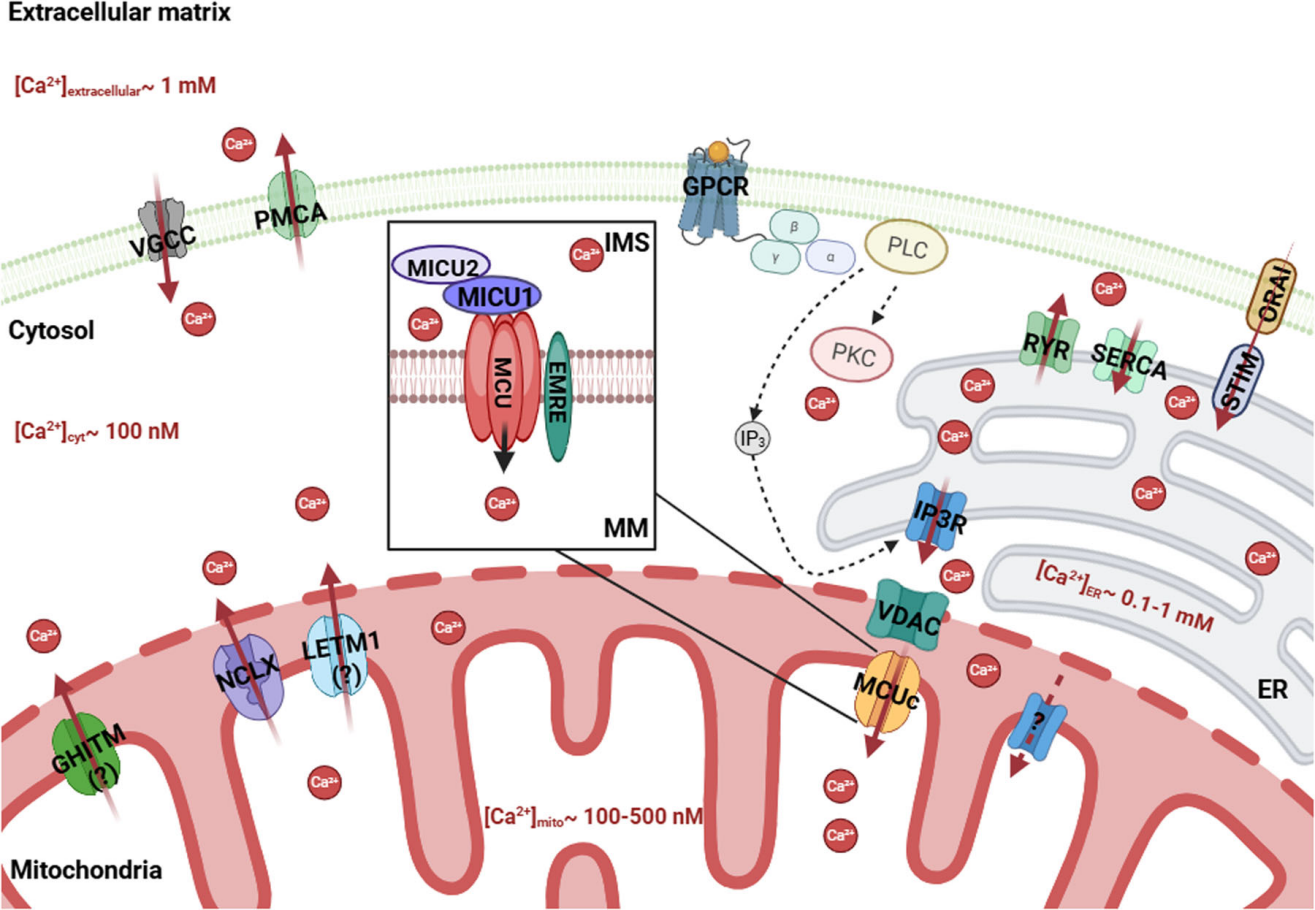
Diagram of Ca^2+^ signaling pathways in a cell. Extracellular [Ca^2+^] is measured on a millimolar scale, whereas cytoplasmic [Ca^2+^] is closer to 100 nM. Calcium flux across the plasma membrane (PM) is mediated by a number of Ca^2+^‐permeable channels and transporters. VGCC, voltage gated Ca^2+^ channels, allow Ca^2+^ entry in a voltage‐dependent manner. PMCA, plasma membrane Ca^2+^ ATPases, export Ca^2+^ from the cytosol at the cost of ATP. GPCR, G‐protein coupled receptors, may couple with the G_q_ G‐protein that activates PLC (phospholipase C) to release diacylglycerol (DAG) and inositol‐1,4,5‐trisphosphate (IP_3_), activating protein kinase C (PKC) and the IP_3_ receptor (IP3R) on the endoplasmic reticulum to increase intracellular Ca^2+^ signaling. The ER has a resting [Ca^2+^] of ~ 0.1–1 mM. IP3R and RYR (ryanodine receptors) mediate efflux from the ER, while the sarco‐endoplasmic reticulum Ca^2+^ ATPase (SERCA) mediates Ca^2+^ uptake. When ER stores are depleted, STIM in the ER and ORAI in the PM coordinate to allow ER refilling. Mitochondrial [Ca^2+^] varies between ~ 100–500 nM at rest. In the outer mitochondrial membrane (OMM), the large‐pore voltage‐dependent anion channel (VDAC) provides a pathway for Ca^2+^ to enter the intermembrane space (IMS). In the mitochondrial inner membrane (IMM), the mitochondrial Ca^2+^ uniporter (MCU) complex mediates Ca^2+^ uptake. Another channel or transporter may contribute to uptake as well, but the molecular identity of such a transporter is not currently known. The Na^+^/Ca^++^ exchanger (NCLX) is known to mediate Ca^2+^ efflux from the mitochondrial matrix (MM) to the IMS, and GHITM and LETM1 have also been suggested to be involved in mitochondrial Ca^2+^ efflux.

Mitochondria are a major target for Ca^2+^ signaling. Mitochondria contain many Ca^2+^ responsive enzymes (Lander et al. 2018; Denton 2009; Denton et al. 1972, 1978; McCormack and Denton 1979) and they are known to have the capacity to act as Ca^2+^ buffers in a number of cellular contexts, including in muscle and immune cells (Andrade et al. 2005; Zweifach and Lewis 1993; Duchen 2000). [Ca^2+^]_m_ is highly regulated by activity, expression, localization, stability, and posttranslational modifications of Ca^2+^‐permeable ion channels and transporters and their regulators (Shanmughapriya et al. 2015; Takahashi et al. 2019; Colussi and Stathopulos 2024; O‐Uchi et al. 2014; Yu et al. 2017; Zhao et al. 2019). [Ca^2+^]_i_ signals of sufficient magnitude and kinetics can propagate into mitochondria regulated in part by the spatial proximity of mitochondrial to regions of elevated [Ca^2+^]_i_ (Tiscione et al. 2021). In regions where the ER and mitochondria are closely apposed at mitochondrial‐associated membrane contact sites (MAMs) (Copeland and Dalton 1959), locally high [Ca^2+^]_i_ provides a mechanism to effect ER‐to‐mitochondria Ca^2+^ transfer, linking receptor‐mediated cell signaling to mitochondrial function (Rizzuto et al. 1993). A graphical representation of these pathways is provided in Figure 1.

### 5.1 Mitochondrial Ca^2+^ Uptake

Mitochondrial Ca^2+^ uptake from the cytoplasm into the matrix involves Ca^2+^ translocation across two membranes. It is generally believed that the VDAC‐containing OMM does not constitute a significant barrier for Ca^2+^ permeation into the intermembrane space (IMS) (Garbincius and Elrod 2022; Benz 1985), whereas the IMM is markedly less permeable. The large IMM membrane voltage, ~ −160 mV, matrix‐side electronegative, provides a powerful thermodynamic driving force for Ca^2+^ entry into the matrix (Bygrave et al. 1971; Vinogradov and Scarpa 1973). The main mode of Ca^2+^ entry into the matrix is the mitochondrial Ca^2+^ uniporter (MCU) ion channel complex. Our understanding of this complex is heavily dependent on both genetic (i.e., knockdown, knockout, and re‐ or overexpression experiments) and pharmacological experiments, which are somewhat hampered by poor pharmacology. While pharmacology for this complex has classically been rooted in nonselective ruthenium‐based compounds (such as Ru360), more targeted inhibitors of this complex have recently been identified by high throughput screenings (De Mario et al. 2021; Di Marco et al. 2020; Arduino et al. 2017), as detailed in the “Targeting mitochondrial Ca^2+^ uptake” section below and recent reviews touching on this subject (Colussi and Stathopulos 2024; Wang et al. 2024). This highly conserved complex consists of the pore forming subunit, MCU (De Stefani et al. 2011), the scaffolding protein essential MCU regulator (EMRE) (Payne et al. 2020; Sancak et al. 2013), and IMS‐localized MICU 1, 2, and 3 proteins (Vais et al. 2020; Csordás et al. 2013; Foskett and Madesh 2014; Liu et al. 2016; Mallilankaraman et al. 2012a; Patron et al. 2019; Perocchi et al. 2010; Plovanich et al. 2013; Wang et al. 2014). It is positively regulated by MCU regulator 1 (MCUR1) (Mallilankaraman et al. 2012b; Vais et al. 2015) and negatively by MCUb, an alternative pore‐forming subunit (Raffaello et al. 2013). MICU proteins associate as dimers consisting of MICU1 with itself, but typically with MICU2, and in some tissues MICU3. MICU proteins are EF‐hand containing Ca^2+^‐binding proteins that associate with the tetrameric MCU pore by binding to MCU and EMRE. In their apo state, MICU1 occludes the entrance to the MCU pore, rendering the channel, and the IMM, impermeable to Ca^2+^. Ca^2+^ binding to MICU proteins induces conformational changes that result in unblocking of the pore, enabling Ca^2+^ permeation through MCU into the matrix in a process referred to as “gatekeeping.” The Ca^2+^ affinities of MICUs ensures that the pore remains occluded until [Ca^2+^]_i_ reaches 1–2 μM (Payne et al. 2020; Vais et al. 2020; Mallilankaraman et al. 2012a; Vais et al. 2015, 2016), concentrations generally not achieved in the bulk cytoplasm during [Ca^2+^]_i_ signaling, but which are present in regions near a source of Ca^2+^, most notably at ER‐mitochondria contract sites. In the absence of MICU1, gatekeeping is abolished and Ca^2+^ flows readily through MCU, resulting in mitochondrial Ca^2+^ overload, with deleterious consequences (Liu et al. 2016; Mallilankaraman et al. 2012a).

Because of the ubiquitous expression of IP_3_R and the MCU complex, and the important role in cellular bioenergetics of ER‐to‐mitochondria Ca^2+^ transfer mediated by these ion channels (discussed below), it was surprising that genetic deletion of the pore‐forming subunit, MCU, has only few overt effects in a whole‐body murine knockout model in pups which survive to birth (Pan et al. 2013). Body sizes of MCU knockout mice are slightly smaller than those of wild‐type mice, and some exercise intolerance is observed (Pan et al. 2013). In contrast, MCU‐null models generated in inbred strains are not viable, though the mechanisms that underly this differential response are currently not understood (Pan et al. 2013; Murphy et al. 2014). MCU‐null cellular models have functional respiration and can grow and proliferate, though these complex phenotypes are generally reduced in comparison to normal‐type controls (Stejerean‐Todoran et al. 2022; Fernandez Garcia et al. 2023; Liu et al. 2020; Weissenrieder et al. 2025; Wang et al. 2022). Notably, Ca^2+^ is present within mitochondria that lack MCU, though these cells lack rapid‐uptake responses to extramitochondrial Ca^2+^ (Fernandez Garcia et al. 2023; Weissenrieder et al. 2025; Wang et al. 2022). Such findings may suggest an as‐yet‐undescribed mode of Ca^2+^ entry into mitochondria, perhaps through reverse flux through Ca^2+^ efflux mechanisms (Garbincius and Elrod 2022; Jiang et al. 2009).

### 5.2 Mitochondrial Ca^2+^ Export

Several proteins have been postulated to contribute to mitochondrial Ca^2+^ export. Most established is the Na^+^/Li^+^/Ca^2+^ exchanger (NCLX, coded for by the *SLC8B1* gene) that exports Ca^2+^ in exchange for Na^+^ or Li^+^ in an electrogenic fashion (Bernardi and Azzone 1982, 1983; Palty et al. 2010). LETM1 (leucine zipper and EF‐hand containing transmembrane protein 1) has been described as an IMM resident Ca^2+^/H^+^ antiporter with impacts on ATP production (Jiang et al. 2009; Piao et al. 2009), although it has also been suggested that LETM1 may indirectly regulate Ca^2+^ efflux by modulating cristae morphology (De Marchi et al. 2014; Nakamura et al. 2020). Growth hormone inducible transmembrane protein (GHITM, also known as TMBIM5), which interacts with LETM1, has been identified as an IMM transmembrane protein that may regulate mitochondrial Ca^2+^ levels through Ca^2+^/H^+^ antiporter activity, with downstream effects on metabolism (Austin et al. 2022; Ren and Schlame 2022; Zhang et al. 2022; Qiang et al. 2017; Reimers et al. 2007). In some situations, mitochondrial Ca^2+^ efflux may occur through the “flickering” action of the mitochondrial permeability transition pore (mPTP) (Bernardi and Petronilli 1996; Ichas et al. 1997). This contrasts with the canonical behavior of mPTP under stress, when it is thought to remain open and promote cell death (Rasola and Bernardi 2011). The molecular composition of the mPTP is poorly understood. Proteins including the ATP synthase (Hunter et al. 1976), the adenine nucleotide transporter (ANT) (Halestrap and Davidson 1990), and cyclophilin D have been posited as complex constituents or regulators (Bernardi and Petronilli 1996; Elrod and Molkentin 2013). While these mitochondrial Ca^2+^ efflux pathways are generally characterized, much of the work in this area has been carried out in the context of normal type cells, particularly in cardiomyocytes. This may limit the interpretation of findings in other contexts, such as in cancer, where typical cellular function often varies from what may be expected from “normal” cells and other cell types due to differences in mutational profiles, epigenetic changes, and downstream expression effects (Hanahan and Weinberg 2011, 2000). To ensure that these pathways are working the same way in the context of cancer, more work is necessary.

### 5.3 Mitochondrial Ca^2+^ Buffering

Within the mitochondrial matrix, Ca^2+^ is heavily buffered by the presence of inorganic phosphates (Chalmers and Nicholls 2003; Wei et al. 2012). Indeed, the bound:free ratio of Ca^2+^ may be as high as 100,000:1 (Chalmers and Nicholls 2003). This high buffering capacity can sometimes influence [Ca^2+^]_i_ signals as well as maintain [Ca^2+^]_m_ at levels sufficient to promote metabolic activity but low enough to prevent cell stress responses, depolarization, and the opening of the mPTP (Colussi and Stathopulos 2024). Mitochondrial Ca^2+^ buffering of cytoplasmic signals is affected by signal amplitudes, frequency, and mitochondrial pH, leading to tissue‐specific differences in behavior (Chalmers and Nicholls 2003; Wei et al. 2012; Csordas 1999; Hajnóczky et al. 1995). Little research regarding mitochondrial Ca^2+^ buffering has been explored in the context of cancer. Thus, it is not known if cancer cells have more mitochondrial Ca^2+^ buffering capacity than their normal‐type counterparts. However, it is likely that phosphate buffering within the mitochondria may contribute to Ca^2+^ handling in cancer as well as in normal cells, and thus it may also contribute to Ca^2+^ handling and cell survival in cases where cells may be exposed to unexpectedly high [Ca^2+^]_i,_ such as when they are treated with ionomycin (Norgard et al. 2021).

## 6 Mitochondrial Metabolism and Ca^2+^

Recent studies have identified a close interrelationship between mitochondrial metabolism and Ca^2+^ signaling. Ca^2+^ can directly and indirectly affect substrate delivery, TCA cycle entry, TCA cycle flux, electron transport chain activity, and ROS generation in mitochondria, with downstream implications for metabolism and cell physiology.

### 6.1 Substrate Entry Into the TCA Cycle

Substrate entry into the TCA cycle is regulated by mitochondrial Ca^2+^ levels. Pyruvate is one of the chief substrates for mitochondrial metabolism. The initial breakdown of glucose into pyruvate by glycolysis occurs in the cytoplasm, though pyruvate may also be formed from amino acids, lactate, and malate by other secondary pathways (Kopperschläger and Kirchberger 1996; Read et al. 2001; Gray et al. 2014; Terrettaz and Jeanrenaud 1990; Green 1936) before entry into the mitochondrial matrix through mitochondrial pyruvate transporters (MPC1, 2, and 3) (Kümmel 1987; Halestrap 1975; Bricker et al. 2012; Herzig et al. 2012). Regulation of this recently discovered import pathway is still unclear. MPC1 expression is repressed in prostate cancer, which is associated with increased glycolysis, growth, and invasion capacity (Li et al. 2016; Wang et al. 2016). These findings are consistent with an increase in aerobic glycolysis in cancer cells. Of note, MPC inhibition increases the expression of Ca^2+^‐regulatory MICU1 and reduces mitochondrial Ca^2+^ uptake in normal hepatocytes, suggesting a link between transporter activity and Ca^2+^ signaling at the IMM (Nemani et al. 2020). Inside the mitochondrial matrix, pyruvate may be dehydrogenated by pyruvate dehydrogenase (PDH) to form acetyl‐Coenzyme A (Ac‐CoA) (Nisman and Mager 1952), or it may enter the TCA cycle through the action of pyruvate carboxylase (PC), which converts it into oxaloacetate, an intermediate for the TCA cycle (Gray et al. 2014; Utter and Keech 1963; Wood and Werkman 1936). [Ca^2+^]_m_ profoundly regulates PDH activity by stimulating the activity of PDH phosphatase to remove inhibitory phosphorylation at multiple sites on the E1α subunit of PDH (Denton 2009; Denton et al. 1972). By disinhibiting PDH, mitochondrial Ca^2+^ stimulates the conversion of pyruvate to Ac‐CoA, enhancing flux through the TCA cycle. The other enzyme that can catalyze entry of pyruvate into the TCA cycle, PC, may promote metabolic plasticity and cellular resilience and metastasis in cancer cells, particularly in cases of glutamine restriction, possibly through effects on lipid metabolism and oxidative stress responses (Cheng et al. 2011; Christen et al. 2016; Kiesel et al. 2021; Phannasil et al. 2017, 2015; Wilmanski et al. 2017; Shinde et al. 2018). PC activity requires the presence of Mg^2+^ (Keech and Barritt 1967) and is regulated by Ac‐CoA concentrations (Chai et al. 2022), and also by [Ca^2+^]_m_, possibly indirectly through effects on PDH (Gray et al. 2014; Dugal and Göpel 1975; Kimmich and Rasmussen 1969). Indeed, the regulatory effects of altered Ca^2+^ concentrations on PC function are stronger in intact cells than in isolated preparations (Gray et al. 2014; Dugal and Göpel 1975; Kimmich and Rasmussen 1969), arguing for an indirect mechanism.

Lipids provide another fuel for the TCA cycle through fatty acid oxidation (FAO), in which 2‐carbon units are progressively removed from fatty acids (FAs) in the mitochondrial matrix to generate Ac‐CoA (Carracedo et al. 2013; Knoop 1904). In certain lipid‐dependent cancers, FAO is critical for cellular growth and survival, of great interest for therapeutic development. Much of our understanding of the relationship between mitochondrial Ca^2+^ homeostasis and FAO comes from studies in muscle tissues (Carracedo et al. 2013; Nieman et al. 2011; Martin‐Perez et al. 2022; Kwong et al. 2018, 2015). In muscle, mitochondrial Ca^2+^ influx through MCU increases FAO when glucose levels are restricted (Kwong et al. 2018). When MCU is knocked out in murine muscle, contractile force and running capacity are reduced, but not dramatically impaired, which has been attributed to an increase in FAO (Kwong et al. 2015; Gherardi et al. 2019). Inhibition of fatty acid uptake and FAO have shown promise in preclinical studies of breast, prostate, glial, and gastrointestinal cancers, among others, but the relationship between mitochondrial Ca^2+^ and FAO has yet to be characterized in cancer cells (Carracedo et al. 2013; Nieman et al. 2011; Tang et al. 2022; Schlaepfer et al. 2014; Gugiatti et al. 2018; Liu et al. 2023a, 2023b; Nandi et al. 2024; Reis et al. 2019; Cirillo et al. 2014).

Glutamine is another major source of carbons for the TCA cycle, particularly in cancer cells. Indeed, many cancers are described as “glutamine addicted” and have a metabolic preference for this substrate (Quek et al. 2022; Wise and Thompson 2010; Eagle 1955). Glutamine is first deaminated by glutaminase (GLS), a mitochondria‐resident protein that produces ammonia and glutamate, which is then deaminated by glutamate dehydrogenase (GDH) to produce α‐ketoglutarate (αKG) (Jin et al. 2023; Yoo et al. 2020). It is unclear if GLS faces outward or inward on the IMM, indicating that mitochondrial metabolism of glutamine may require either glutamine or glutamate transporters, the identities of which remain unknown (Scalise et al. 2017; Gyimesi and Hediger 2020). The effects of mitochondrial or cytoplasmic Ca^2+^ on GLS are not currently characterized, though Ca^2+^ reportedly stimulates GDH function in *Bacillus* and *Arabidopsis (*Grzechowiak et al. 2023; Meng et al. 2016). Interestingly, a high‐throughput screen for GDH inhibitors identified multiple Ca^2+^ modulators, including a Ca^2+^ channel blocker and a calmodulin antagonist, suggesting that GDH function may be closely regulated by upstream changes in Ca^2+^, though the compartments which may be involved are not currently characterized (Li et al. 2007). While inhibitors of glutamine metabolism exist, their effects on mitochondrial Ca^2+^ signaling are poorly understood outside of the context of mPTP (Wise and Thompson 2010; Yoo et al. 2020). One such inhibitor, l‐DON (6‐diazo‐5‐oxo‐l‐norleucine), induces mPTP, enhancing Ca^2+^ overload induced by calcium carbonate co‐delivered in nanoparticles (Chen et al. 2025).

In addition to glutamine, other amino acids may also impact cellular metabolism in the context of altered mitochondrial Ca^2+^ homeostasis. Cystine has been identified as a critical, targetable metabolic vulnerability in pancreatic cancer cells lacking MCU (Wang et al. 2022). Metabolic dependencies for serine (Maddocks et al. 2013; Tajan et al. 2021), asparagine (Knott et al. 2018; Krall et al. 2016; Yoo et al. 2024; Zhang et al. 2014), and other amino acids (Lieu et al. 2020; Wei et al. 2020) have been reported in a number of different cancer types and may be present or exacerbated when mitochondrial Ca^2+^ fluxes are altered. However, very little work has been done in the area, and tissue specific effects may be expected.

### 6.2 TCA Cycle Flux

While PDH is considered a gatekeeper for acetyl‐CoA and a rate‐limiting step for flux of glucose carbons through the TCA cycle, the cycle is also subject to a high degree of internal regulation. This regulation can occur through alterations in enzymatic protein pools, posttranslational modifications, and [Ca^2+^]_m_ (reviewed in [Arnold and Finley 2023]). Notably, increasing [Ca^2+^]_m_ reduces the Michaelis constant (K_m_) of both isocitrate dehydrogenase (IDH) and αKG dehydrogenase (αKGDH) (Denton 2009; Denton et al. 1978; McCormack and Denton 1979; Rutter and Denton 1988). In IDH, high [Ca^2+^] increases conversion of isocitrate to αKG and CO_2_ (Denton 2009; Rutter and Denton 1988, 1989a, 1989b). The magnitude of the effect of [Ca^2+^]_m_ is altered by the ATP/ADP ratio, with higher sensitivity in low ATP/high ADP conditions. The effect of ATP/ADP on Ca^2+^ sensitivity is more profound in IDH than in αKGDH or PDH, providing an additional layer of regulation wherein mitochondrial Ca^2+^ influx may more strongly stimulate IDH in low‐energy conditions (high ADP, low ATP) to promote the downstream action of the ETC (Rutter and Denton 1988, 1989a). Downstream of IDH, αKGDH converts αKG to succinyl‐CoA, converting NAD^+^ to NADH in the process. Increased [Ca^2+^]_m_ reduces the K_m_ of αKGDH in a similar manner to that observed in IDH, though αKGDH is more sensitive to NADH/NAD^+^ and succinyl‐CoA/CoA ratios than ATP/ADP ratio (Denton 2009; Rutter and Denton 1988; Vatrinet et al. 2017). Thus, IDH appears to “turn on” when energy pools are low and [Ca^2+^]_m_ is high, whereas αKGDH activity is increased in response to increases in [Ca^2+^]_m_ but serves as a “brake” in conditions where the downstream products of αKGDH build up. While other proteins in this cycle are not considered to be Ca^2+^ ‐sensitive, they are regulated by substrate and product concentrations which are, in turn, altered by the regulation of the Ca^2+^‐dependent dehydrogenases, PDH, IDH, and αKGDH. Thus, TCA cycle flux, and potentially metabolite pools, increases in response to Ca^2+^ influx into the mitochondrial matrix.

### 6.3 Electron Transport Chain

Mitochondria are critical for the generation of ATP, which is used to power many biological processes. OXPHOS, carried out in the mitochondria, generates 15 times the amount of ATP per molecule of glucose compared with glycolysis alone (Lehninger et al. 2013). OXPHOS relies on the TCA cycle to provide reducing equivalents to the ETC to create the proton motive force that powers the ATP synthase (Simon et al. 2008). ATP availability is critical for all cells, and it may have special importance in cells which are motile or secretory, as these processes are ATP‐driven (Doerschug and Chrispeels 1970; Kruse and Bornstein 1975). Crucially, these processes may be upregulated in cancer cells, particularly those engaged in active metastasis (Robinson et al. 2019). Ca^2+^ and ETC function are tightly interrelated. ETC function promotes Ca^2+^ influx by increasing the negative membrane potential of the IMM (Alberts 2008; Duchen 2000; Poburko et al. 2011). In turn, high [Ca^2+^]_m_ stimulates the TCA cycle to provide substrates for the ETC (Glancy et al. 2013). Reducing the functionality of the ETC by knocking down *NDUFAF3* (NADH dehydrogenase [ubiquinone] 1 alpha subcomplex assembly factor 3) and *SDHB* (succinate dehydrogenase iron sulfur subunit B) reduces Ca^2+^ influx and efflux, independent of the mitochondrial membrane potential (Jaña et al. 2019). Similarly, in normal murine pancreatic β cells, knockdown of complex III altered redox status and reduced Ca^2+^ signals downstream of glucose stimulation (Lang et al. 2023). Ca^2+^ may also affect the function of F_1_F_0_ ATPase (Complex V, ATP synthase) through impacts on ion balance, substrate availability, phosphorylation, and transcriptional regulation (Territo et al. 2000; Williams et al. 2015).

### 6.4 Reactive Oxygen Species

ROS are generated by metabolic processes that lead to energy production, including by the TCA cycle (notably during the conversion of αKG to succinyl‐CoA) (Starkov et al. 2004; Tretter and Adam‐Vizi 2004) and the ETC (Liu et al. 2002; Turrens et al. 1985; Raimondi et al. 2020). Ca^2+^ signaling and ROS are profoundly interrelated. While high [Ca^2+^]_m_ increases flux through these metabolic pathways, ROS can cause alterations in both metabolism (Tabatabaie et al. 1996) and Ca^2+^ handling (Görlach et al. 2015; Dong et al. 2017a) by altering protein function. Some studies have suggested that observed increases in ROS in these cases may be due to alterations in mitochondrial membrane potential (Suski 2012). In isolated mitochondria, high [Ca^2+^]_m_ can lead to the opening of the mitochondrial permeability transition pore (mPTP) and a rapid increase in ROS generation (Görlach et al. 2015; Baumgartner et al. 2009; Sousa et al. 2003; Cadenas and Boveris 1980; Araki et al. 1992; Hansson et al. 2008). Antioxidant treatment inhibits mPTP opening and may reduce Ca^2+^ levels in some contexts, potentially reducing toxicity (Rajesh et al. 2003; Salimi et al. 2021). On the other hand, Ca^2+^ signaling can reduce ROS signaling in certain tissues and contexts, such as in isolated brain mitochondria and in the case of high membrane potential (Görlach et al. 2015; Starkov et al. 2002; Adam‐Vizi and Starkov 2010). These findings suggest that here, too, Ca^2+^ signaling can play multiple roles in regulation, with great potential for tissue and context specificity.

## 7 Mitochondrial Ca^2+^ in Cancer

Given the well‐known importance of Ca^2+^ in protein regulation, metabolism, and signaling pathways in the cytoplasm and the highly dynamic regulation of these signals to mitochondria, it is not surprising that mitochondrial Ca^2+^ homeostasis can significantly affect cancer development, growth, and survival. Indeed, many studies have shown linkages between Ca^2+^‐modulating and ‐modulated proteins in mitochondria and cancer‐related phenotypes. Importantly, some of these studies have revealed that altered Ca^2+^ signaling to mitochondria may have differential effects in malignant models versus normal‐type controls (Fernandez Garcia et al. 2023; Cárdenas et al. 2016). We recently reported that compared with transformed cells, immortalized murine fibroblasts have reduced mitochondrial Ca^2+^ uptake rates, associated with reduced MCU expression levels and oxygen consumption rates (Fernandez Garcia et al. 2023). Inhibition of ER‐to‐mitochondrial Ca^2+^ by knockdown of *MCU* or *ITPR1/3* elicited a nearly three‐fold increase in LDH release, a measure of cytotoxicity, in transformed human fibroblasts as compared to immortalized ones (Cárdenas et al. 2016). These findings suggest that a therapeutic window may exist for the targeting mitochondrial Ca^2+^ flux pathways, despite their ubiquity.

A growing body of work has strongly indicated that components of the MCU complex, particularly MCU and MICU1, may have critical roles in multiple cancers. In human patients, higher MCU expression is associated with poor survival outcomes in breast, liver, and pancreatic cancers (Stejerean‐Todoran et al. 2022; Weissenrieder et al. 2025; Tosatto et al. 2016), and MCU expression is positively correlated with tumor size and metastatic potential in breast and pancreatic cancer (Weissenrieder et al. 2025; Wang et al. 2022; Tosatto et al. 2016; Curry et al. 2013). MCU affects breast cancer progression and metastasis through altered ROS generation and HIF1α (hypoxia inducible factor 1 alpha) activity (Tosatto et al. 2016), and silencing MCU may increase caspase‐independent cell death in these models (Curry et al. 2013). In a murine organoid model of pancreatic cancer development, MCU complex components *Mcu*, *Micu1* and *Smdt1* (EMRE) transcripts are upregulated with increasing malignancy (Weissenrieder et al. 2025). Alterations in expression of MICU1 and MICU2 downstream of HINT1 (histidine triad nucleotide‐binding protein 1) activity have been linked to altered sensitivity to gemcitabine‐induced apoptosis (Chen et al. 2017; Xie et al. 2019). Here, reducing *MICU1/2* expression may increase apoptotic sensitivity by allowing more Ca^2+^ influx through MCU, leading to mitochondrial Ca^2+^ overload (Chen et al. 2017). Similarly, exogenous *EMRE* overexpression in human pancreatic adenocarcinoma (PDAC) cells reduced cell proliferation, altered mitochondrial dynamics, and increased apoptosis (Xie et al. 2019). Finally, *MCUR1* expression improves cellular survival through a ROS and p53 (tumor protein p53) mediated mechanism in liver cancer, as observed in both models of overexpression in low‐expressing cells and knockdown experiments in high‐level expressers (Ren et al. 2018). Such findings suggest that expression of MCU complex‐related genes may be modulated by expression levels of other complex members and/or have cell‐type dependent effects.

The MCU channel complex appears to exert at least some of these cancer‐related effects by affecting the activities of metabolic pathways. In pancreatic cancer models, MCU expression promotes dependency on exogenous cystine, suggesting that mitochondrial Ca^2+^ uptake may promote changes in metabolic substrate preferences (Wang et al. 2022). Supporting the notion that MCU activity may regulate preferential use of metabolic substrates, MCU knockout in a transformed fibroblast model is associated with an increase in glucose uptake and aerobic glycolysis, with increased fractional incorporation of glucose‐derived carbons in pyruvate, lactate, and some amino acids (Fernandez Garcia et al. 2023). Similarly, *MICU1* expression increases glycolytic flux and promotes chemoresistance in ovarian cancer (Chakraborty et al. 2017; Li 2022; Rao et al. 2020; Zhao et al. 2023a). Importantly, MCU expression also promotes mitochondrial biogenesis in colorectal cancer, which may promote mitochondrial metabolism (Liu et al. 2020). Interestingly, in some cancers, including melanoma, kidney, and colorectal cancer, the opposite trend is seen, with high MCU expression correlated with *increased* patient survival (Stejerean‐Todoran et al. 2022). Bogeski et al. have shown that MCU knockdown reduces primary melanoma lesion size but increases lung metastases in melanoma xenograft models through a mechanism influenced by redox status (Stejerean‐Todoran et al. 2022). In many other cancer types, including lung, brain, and ovarian malignancies, MCU expression does not significantly impact overall survival in human patients (Stejerean‐Todoran et al. 2022). The differences between these groups are as yet unclear but do not appear to involve the expression of driver mutations in *BRAF* or *NRAS* (Stejerean‐Todoran et al. 2022).

Mitochondrial Ca^2+^ efflux through reported ion exchangers such as NCLX, LETM1, and GHITM has also been implicated in metabolic and cancer phenotypes. NCLX, the Na^+^/Ca^2+^ exchanger, has been reported to regulate glycolysis and autophagy in normal‐type cells (Cabral‐Costa et al. 2023; Taha et al. 2024; Tarasov et al. 2012), while LETM1, a proposed Ca^2+^/H^+^ antiporter, appears essential for normal glucose metabolism (Jiang et al. 2013). LETM1 appears to be increased in several cancer subtypes and is linked to cancer stem cell‐like populations (Piao et al. 2009; Che et al. 2021; Ji and Hu 2020; Li et al. 2020; Piao et al. 2019; Wang et al. 2020a; Zhang et al. 2020; Zhou et al. 2020). Conversely, NCLX is reduced in colorectal cancer, associated with mitochondrial Ca^2+^ overload, increased ROS production, and reduced cell growth and tumor size (Pathak et al. 2020; Guéguinou et al. 2022), as well as with activating HIF1α activation and enhanced metastasis (Pathak et al. 2020; Guéguinou et al. 2022). GHITM (also known as TMBIM5), another proposed Ca^2+^/H^+^ antiporter, is similarly downregulated in renal cancer, and its overexpression inhibited proliferation and migration in a cell line model (Huang et al. 2024). Thus, activation of mitochondrial efflux pathways may reduce growth of tumors, but potentially at the expense of increased metastasis or tumor‐stem‐cell‐like properties. Given that reduced mitochondrial Ca^2+^ influx by MCU inhibition and increased Ca^2+^ efflux are associated with smaller tumors but increased metastatic potential, reduced [Ca^2+^]_m_ may regulate a phenotypic switch between proliferative and metastatic states. Indeed, other studies have suggested that [Ca^2+^]_m_ may impact cellular plasticity in other contexts, with MCU knockout reducing basal propensity for epithelial‐to‐mesenchymal transition (EMT) in PDAC, at least partially through alterations in cellular secretomic capacity (Weissenrieder et al. 2025). PDAC cells lacking MCU were able to undergo EMT, but required exogenous pressure to do so (through exogenous TGFβ administration or stable expression of the EMT transcription factor, Snail). Another study has suggested that global Ca^2+^ elevations induced by ionomycin treatment may induce a hybrid, partial EMT state in PDAC (Norgard et al. 2021). Finally, MCUR1 may facilitate EMT in hepatocellular carcinoma (Ren et al. 2018).

Taken together, these studies suggest that regulation of mitochondrial Ca^2+^ flux by IMM‐resident ion channels and transporters may be critical for both survival and apoptosis, leading to cell death when Ca^2+^ levels deviate from the preferred range of a given cell type. The “Goldilocks zones” of [Ca^2+^]_m_ and [Ca^2+^]_m_ dynamics in cancer are not known and are likely to be cell‐type specific (Garbincius and Elrod 2022; Colussi and Stathopulos 2024). Mechanisms linking mitochondrial Ca^2+^ influx to cancer cell outcomes appear to involve pro‐ and anti‐ apoptotic proteins, ROS generation, and metabolism, though much remains undefined in this active area of research. Potential targeting of this axis in human patients in the future may require precision medicine techniques, given that different cancers may have respond differently to MCU targeting. Future work is therefore critical to inform appropriate preclinical model development and analysis and elucidate the potential for targeting this axis in human cancers.

## 8 Mitochondrial Ca^2+^ as an Anticancer Target

Mitochondrial Ca^2+^ homeostasis may represent a potential anticancer target with an as‐yet underappreciated importance and potential for selectivity for cancer cells over normal cells (Fernandez Garcia et al. 2023; Cárdenas et al. 2016). It is therefore not surprising that there is interest in development of modulators of mitochondrial Ca^2+^ channels for potential anticancer use, as mitochondria are critical for cancer cell health and function (Fernandez Garcia et al. 2023; Cárdenas et al. 2016; Pathak et al. 2020; Sterea and El Hiani 2020; Weissenrieder et al. 2025; Miao et al. 2023). In addition, controlling mitochondrial Ca^2+^ uptake and efflux may both be useful in both research and treatment paradigms in a variety of other diseases, including metabolic and cardiac dysfunctions, expanding the potential use cases for chemical modulators of these channels (Pan et al. 2013; Kwong et al. 2015; Allen and Tessem 2022; Alves‐Figueiredo et al. 2024; Bround et al. 2024; Liu 2020; Petersen et al. 2023; Weiser et al. 2021). Small molecule modulators of mitochondrial Ca^2+^ uptake and efflux transporters, including MCU, MICU, and NCLX, have been developed and are in use for preclinical modeling in cancer, neurodegeneration, and cardiac research studies (discussed below). As yet, however, none of these have progressed to use in the clinic. Existing compounds are often limited by violations of Lipinski's rule of 5, delivery issues, lack of specificity, and poor efficacy (Lipinski et al. 2001). Lipinski's rule of five states that a small molecule is most likely to present a suitable candidate for drug development if it: (1) is ≤ 500 kDa in size, (2) has a partitioning coefficient (clogP) of ≤ 5 (i.e., is not too lipophilic), (3) has no more than five hydrogen bond donors, and (4) has no more than 10 hydrogen bond acceptors (Lipinski et al. 2001). Other research has suggested that targeting upstream regulators of expression by transcription factors such as HIF1α (Tosatto et al. 2016), or miRNA, such as miR‐25 (Marchi et al. 2013) and miR‐17 (Yu et al. 2017), may represent a viable mechanism for successful control of Ca^2+^ flux at the IMM (reviewed in [Colussi and Stathopulos 2024]).

### 8.1 Targeting Mitochondrial Ca^2+^ Uptake

Targeting mitochondrial Ca^2+^ uptake may be useful to limit tumor cell growth (Fernandez Garcia et al. 2023; Liu et al. 2020; Wang et al. 2022; Tosatto et al. 2016), and it is also of interest in ischemia/reperfusion injury (Xue et al. 2017; Li et al. 2021). As such, development of selective small molecule inhibitors of this pathway has become an area of active research over the past decade (Di Marco et al. 2020; Arduino et al. 2017). Early work focused on the use of ruthenium‐based compounds, such as Ru360 (Matlib et al. 1998) and Ru265 (Woods et al. 2019). These oxygen‐ or nitrogen‐ bridged ruthenium compounds bind to and inhibit the MCU complex with high (low nanomolar) affinity and interact directly with the DIME motif, a critical motif located at the pore entrance of the MCU complex (Novorolsky et al. 2020; Woods et al. 2021). These compounds are valuable for mechanistic research, particularly in isolated mitochondrial preparations. However, they have many off‐target effects, stability issues, and limited permeability at the plasma membrane related to their size, charge, complexity, and tendency to bind membranes (Colussi and Stathopulos 2024). While they have been deployed in rodent models, off‐target, pro‐convulsant activity precludes use in human patients (Xu et al. 2024b).

Other compounds that modulate mitochondrial Ca^2+^ uptake have been developed or identified by high‐throughput screenings (Di Marco et al. 2020; Arduino et al. 2017; Kon et al. 2017). One of these, DS16570511, was initially described as a cell‐permeable, specific inhibitor of the MCU complex with an apparent IC_50_ of ~ 7 μM (Kon et al. 2017), but later work showed that this compound depolarizes the mitochondrial membrane potential in a concentration dependent manner, independently of MCU expression (Payne et al. 2019). Decreased membrane potential inhibits mitochondrial Ca^2+^ uptake in the presence of functional MCU, as well as affecting mPTP activity and ATP synthesis, potentially affecting multiple downstream pathways (Colussi and Stathopulos 2024). This compound is also of limited interest for further drug development due to Lipinski violations, being > 500 Daltons with an unfavorably high lipophilicity. Similarly, the antimalarial agent, dihydroartemisinin, has shown promise as an anticancer agent and reduces Ca^2+^ flux through the MCU complex, though it appears that this inhibition may be indirect and, like DS16570511, related to altered mitochondrial membrane potential (Chen et al. 2021; Zheng et al. 2022; Zhu et al. 2018). Unfortunately, this compound also has low oral bioavailability, and it induced anorexia in tumor‐bearing canines (Hosoya et al. 2014).

Mitoxantrone was identified in a high‐throughput screen for MCU complex inhibitors (Arduino et al. 2017). It has a apparent IC50 of ~ 10 μM for MCU and is an FDA‐approved anthracinedione chemotherapeutic agent which may be used to treat advanced prostate cancer, acute non‐lymphocytic leukemia, and, in some cases, multiple sclerosis (Faulds et al. 1991). Unfortunately, mitoxantrone has a much higher affinity and activity at other substrates, including PIM1 kinase ( ~ 50 nM) and topoisomerase II ( ~ 100 nM – 1 μM) (Atwal et al. 2019; Evison et al. 2016). It has been linked to cardiotoxicity, mitochondrial membrane potential hyperpolarization, and alterations in ETC activity and ATP synthesis (Rossato et al. 2014, 2013; Varga et al. 2015). A structural analog of mitoxantrone, pixantrone, also binds to the DIME motif of MCU in a similar manner to mitoxantrone, albeit with a reduced affinity (~ 17 μM), but appears to have reduced cardiotoxicity and increased antitumor effects (Cavalletti et al. 2007). Its effects on MCU function, mitochondrial membrane potential and ETC activity are less well understood than mitoxantrone.

In another high‐throughput screen, 44,000 compounds were screened to identify modulators of MCU complex activity (Di Marco et al. 2020). Two compounds, MCU‐i4 and MCU‐i11, were identified that reduced agonist‐induced mitochondrial Ca^2+^ influx by ~ 25%–50% at 10 μM of compound. MCU‐i4 and MCU‐i11 have apparent K_D_s in the single micromolar range, and their effect requires the expression of MICU1 but not MICU2. Their effects on mitochondrial Ca^2+^ uptake are likely attributable to their binding to MICU1. Perhaps because they function at a regulatory protein rather than at the pore, these compounds have a relatively modest effect on [Ca^2+^]_m_ (Di Marco et al. 2020). These compounds might therefore be expected to provide a modulatory effect rather than significant inhibition in intact cells. Similarly, benzethonium, a quaternary ammonia salt with antiseptic properties, reduces agonist‐induced mitochondrial Ca^2+^ uptake in HeLa cells, with concomitant reductions in subsequent ROS generation (De Mario et al. 2021). When applied to MDA‐MB‐231 breast cancer cells, this results in modestly reduced clonogenic capacity, wound healing activity, and cell proliferation (De Mario et al. 2021). This compound also reduced tumor growth by ~15% in a murine in vivo xenograft model of FaDu cells at 5 mg/kg (Yip et al. 2006). In contrast, the natural product oleuropein, as well as the FDA‐approved antifungal agent, amorolfine, have been shown to enhance MCU function (De Mario et al. 2021; Gherardi et al. 2025).

Other work has elucidated potential small molecule inhibitors that may impact mitochondrial Ca^2+^ uptake indirectly, though many of these have not been thoroughly explored in cancer models. For instance, metformin, which is approved for use in the treatment of type II diabetes, reduces expression of *MCU* and *MICU1* and [Ca^2+^]_m_ in some models (Angebault et al. 2020). This effect may be related to altered ETC activity or changes in interactions between the ER and mitochondria (Angebault et al. 2020). In other cancer models, high concentrations of metformin (5 mM) induced ER stress, mitochondrial Ca^2+^ overload, and mitochondrial swelling (Loubiere et al. 2017). These effects are expected to be off‐target and may be of limited biological relevance since circulating plasma concentrations of only 10–40 μM are achieved in human and animal models treated with metformin (He and Wondisford 2015; Wilcock and Bailey 1994). Salsolinol (Wen et al. 2019), a chemical relative of dopamine, and Ginkgolide K (Liu et al. 2022), a compound isolated from Gingko biloba extract, also affect MCU expression and [Ca^2+^]_m_ through poorly characterized, likely indirect, mechanisms. A natural product, astragaloside‐IV (from *Astragalus membranaceus*) appears to modulate MCU in such a fashion to reduce over‐activation of MCU which might result in mitochondrial Ca^2+^ overload (Dong et al. 2017b). MCU complex activators have also been identified, including kaempferol (Chitturi et al. 2019) and aloe‐emodin (from *aloe vera*) (Gao et al. 2021). These may be of interest preclinically and may also promote mitochondrial Ca^2+^ overload and cell death in some models, though these areas have not been explored thoroughly.

Ca^2+^ signaling from the ER to mitochondria is canonically activated by signals emanating from Gq‐coupled GPCRs. The Gq subunit, encoded by *GNAQ*, is often mutated in some forms of cancer in which the enzyme spends increased time in the active, GTP‐bound state (Van Raamsdonk et al. 2009; Annala et al. 2019; Bos et al. 2007; Campbell and Smrcka 2018). This may represent a target to reduce pathological Ca^2+^ signaling (Annala et al. 2019). In addition, Gq‐coupled receptors themselves may present opportunities for anticancer targeting, either through the control of ligand availability or through receptor modulation by small molecules. Notably, GPR30, a GPCR tied to the progression of multiple cancers, was recently reported to be coupled to Gq (Urban et al. 2023), though research was complicated by wide variations in the localization of a GFP‐fusion protein between cell lines. The ligands of this GPCR are hotly contested and may or may not include estrogen (Urban et al. 2023; Ariazi et al. 2010; Tutzauer et al. 2021; Lee et al. 2023; Natale et al. 2020). Finally, modulation of ER stores through alterations in store operated Ca^2+^ entry, ryanodine receptor activation, and SERCA function may provide another method for upstream regulation of Ca^2+^ exposure to mitochondria (Panda et al. 2021). Finally, receptor tyrosine kinases (RTKs) may also affect these signals by increasing phospholipase C activation and thus IP3 generation (Wilde and Watson 2001). Indeed, cardiac toxicities associated with the use of RTK inhibitors are suspected to be related to altered intracellular Ca^2+^ signaling (Barr et al. 2014; Shyam Sunder et al. 2023) and mitochondrial dysfunction (Shyam Sunder et al. 2023; Kerkelä et al. 2006). Studies have shown that RTK inhibitors such as imatinib and dasatinib can negatively impact mitochondrial function in myotubes and myoblasts, resulting in altered mitochondrial membrane potential, oxidative stress, reduced ATP generation, and reduced complex I activity (Bouitbir et al. 2020). This study was limited by the use of supraphysiological concentrations of compounds (20 μM imatinib and 1 μM dasatinib), and Ca^2+^ signaling was not directly measured. However, these phenotypes would be consistent with altered Ca^2+^ signaling in the mitochondria, and these compounds do not appear to have direct off‐target effects on mitochondrial function (Will et al. 2008).

### 8.2 Targeting Mitochondrial Ca^2+^ Efflux

On the other side of the coin, mitochondrial Ca^2+^ homeostasis may also be modulated through alterations in Ca^2+^ efflux. Modulators of efflux may therefore also be of interest for preclinical studies and/or therapeutic development. As such, it is not surprising that inhibitors of NCLX have been developed. CGP37157 is a benzothiazepine derivative of clonazepam that blocks NCLX with an IC_50_ of ~ 360 nM in isolated mitochondria (Cox et al. 1993). However, it has also been shown to affect other Ca^2+^ channels, including sarco/endoplasmic reticulum Ca^2+^ ATPase (SERCA), CALHM channels, ryanodine receptors, and voltage‐gated Ca^2+^ channels (Moreno‐Ortega et al. 2015; Neumann et al. 2011; Ruiz et al. 2014). A related inhibitor of mitochondrial Na^+^/Ca^2+^ exchange, ITH12575, has an IC50 of ~ 690 nM in HeLa cells (De Marchi et al. 2014). Recent work in colorectal cancer models has also identified curcumin as an inhibitor of mitochondrial Ca^2+^ efflux (Guéguinou et al. 2022). In this study, both curcumin and CGP37157 reduced cell viability, xenograft growth, and metabolic activity of HCT116 colorectal cancer cells. These alterations were associated with mitochondrial Ca^2+^ overload, increased ROS generation, and slowed progression through the cell cycle. Curcumin interacts with many other targets, including a variety of proteins and metal ions (Baum and Ng 2004; Gupta et al. 2011). Thus, available modulators for mitochondrial Ca^2+^ efflux, like those available for mitochondrial Ca^2+^ uptake, are nonselective and therefore pose problems for translation to the clinic.

### 8.3 Chemotherapeutic Synergy

Numerous studies have implicated mitochondrial Ca^2+^‐related pathways in the regulation of apoptotic sensitivity, suggesting that modulation of mitochondrial Ca^2+^ homeostasis could impact chemotherapeutic responses (Duchen 2000; Chen et al. 2017; Chakraborty et al. 2017; Dubois et al. 2020). Indeed, multiple preclinical studies have observed alterations in chemotherapeutic sensitivity when IMM‐resident, Ca^2+^‐permeable channels are modulated (Chen et al. 2017; Chakraborty et al. 2017; Guéguinou et al. 2022). Genetic knockdown of MCU increased cisplatin response in esophageal squamous cell carcinoma (Miao et al. 2023). Similarly, MCU knockout sensitized pancreatic cancer cells to both gemcitabine and 5‐fluorouracil (Weissenrieder et al. 2025). Such effects may be related to the induction of apoptosis downstream of DNA damage induced by these agents, which may be exacerbated by a reduction of nucleotide availability downstream of reduced TCA cycle function, though this was not directly explored. Reduction of Ca^2+^ flux across the IMM may also increase sensitivity to ferroptosis and necrosis in certain cancer models, providing alternative pathways to cell death under cellular stress which may augment responses to chemotherapeutics in an additive manner (Wang et al. 2022; Cárdenas et al. 2016). However, in melanoma, MCU knockdown induced resistance to immunotherapy and ferroptosis (Stejerean‐Todoran et al. 2022). These differences underscore the importance of tissue of origin in responses to mitochondrial Ca^2+^ modulation, as some types of cancer may better resist therapeutic treatments under the same conditions that may sensitize others.

Pharmacological targeting of cytoplasmic Ca^2+^ signaling could be an indirect approach to target mitochondrial Ca^2+^ homeostasis in cancer. Many Ca^2+^ channel blockers are used clinically to treat cardiovascular diseases. Examination of the relationship between use of Ca^2+^ channel blockers and cancer risk has not revealed a consistent correlation, though some studies have suggested that the blockers may induce a small increase in cancer risk for certain types of cancer or in certain populations (Copland et al. 2021; Fong et al. 2024; Lin et al. 2023; Olsen et al. 1997; Pahor 1996a, 1996b; Rosenberg 1998). In a recent retrospective population study of pancreatic cancer patients undergoing resection with curative intent, Ca^2+^ channel blocker treatment was associated with improved survival outcomes over neoadjuvant therapy alone (Fong et al. 2024). No effect was observed in patients treated with Ca^2+^ channel blockers without neoadjuvant therapy, suggesting that altered Ca^2+^ fluxes may impact cellular responses to chemotherapeutic treatment in PDAC. This is consistent with findings that Ca^2+^channel blockers, amlodipine or nifedipine, sensitized murine pancreatic cancer models to gemcitabine (Principe et al. 2022).

## 9 Conclusions

Modulation of mitochondrial Ca^2+^ homeostasis presents a unique opportunity to control cancer cell metabolism and survival in a variety of different malignancies. This potential is not without complications, as research has shown that effects on the pathways involved in mitochondrial Ca^2+^ metabolism are context‐ and tissue‐ dependent. In some models of cancer, blocking mitochondrial Ca^2+^ influx may reduce cell metabolism, growth, and metastasis (Fernandez Garcia et al. 2023), while sensitizing to currently used chemotherapeutic agents (Chen et al. 2017; Weissenrieder et al. 2025). In other cancers like melanoma, blocking mitochondrial Ca^2+^ influx may induce resistance to commonly used therapeutics and increase metastasis (Stejerean‐Todoran et al. 2022). The cause of these differences is yet to be elucidated, and it is a critical area for future research.

A major conclusion that can be drawn from the current body of literature is that [Ca^2+^]_m_ levels may need to be maintained within cells within a set range. If they are too low, the metabolic machinery will fail to function; too high, and [Ca^2+^]_m_ overload will induce ROS, mPTP opening, and cell death. This acceptable range may vary based on cell type, nutrient availability, buffering capacity, and cell signaling, among other potential inputs. Additionally, differences in the expression and function of mitochondrial Ca^2+^ transporters may restrict or amplify sensitivity to signals. For example, blockade of mitochondrial Ca^2+^ efflux might increase the risk of mitochondrial Ca^2+^ overload during cell stimulation, whereas reductions in Ca^2+^ influx might restrict metabolic responses.

While there is a clear relationship between mitochondrial Ca^2+^ homeostasis and cancer cell survival and metabolism, less obvious are the mechanisms involved. Ca^2+^ can enhance enzymatic activities in the TCA cycle, stimulate the ETC, and increase ATP generation, while enhanced ETC activity can influence [Ca^2+^]_m_ through effects on mitochondrial membrane potential. Many studies that have elucidated these relationships have been carried out in normal‐type cells, rather than in malignant and tumorigenic cells, and a full characterization of these relationships in the context of cancer is crucial to determine if these processes represent targetable vulnerabilities. If pathways involved in mitochondrial Ca^2+^ homeostasis are to be pursued as potential anticancer therapeutic targets, understanding the driver mutations, metabolic contexts, and gene expression alterations which may underly these relationships may enable patients to be striated, avoiding possibilities of applying treatments which may have negative effects. Generating a thorough understanding of these processes will require a combination of in vitro and in vivo research, and an in‐depth analysis of human data sets.

Our understanding of the effects of mitochondrial Ca^2+^ on cancer cell metabolism, growth, and survival are limited by the lack of selective small molecule modulators for the channels and exchangers involved in mitochondrial Ca^2+^ homeostasis. Ideal modulators would be highly selective, with high affinity, limited lipophilicity, and an ability to cross the plasma membrane easily, with limited off‐target effects and high potential for cancer cell delivery. To date, high‐throughput screens have failed to lead to the development of compounds with therapeutic potential. Cryo‐electron microscopic structures of the MCU channel complex have been recently obtained, providing an opportunity for drug development by molecular modeling (Fan et al. 2020; Wang et al. 2020b). Mitochondrial metabolism is strongly regulated by Ca^2+^ signaling cascades that emanate from upstream signals at the plasma membrane, which might also present potential targets for modulation as cancer treatments.

Elucidation of the relationships between mechanisms involved in mitochondrial Ca^2+^ homeostasis and cancer cell biology has been somewhat limited by the preclinical models that are currently available. Lacking robust pharmacological tools, many cancer models have relied on stable Cre‐, CRISPR‐, or shRNA‐ mediated knockdown or knockout genetic models, which may be complicated by compensatory mechanisms. Acute models are necessary to clarify what effects pharmacological modulation may have on cancer models in a more treatment‐adjacent paradigm. This could be provided by the use of inducible CRISPR knockout systems for cell‐line models or Flp‐mediated genetic knockout in murine systems (Schönhuber et al. 2014). Similarly, much of our knowledge has been generated in studies of cells in 2D culture or in subcutaneous xenograft models. 2D cultures are typically grown in highly nutritious media which may not adequately model the nutrient availability within the tumor microenvironment (Sullivan et al. 2019), and the oxygenation of the environment is often higher than that expected in tumor tissues, which are often highly hypoxic (Chen et al. 2023b). These conditions may thus mask metabolic dependencies that may be of biological importance in vivo. Some of these limitations could possibly be ameliorated by the use of 3D models, hypoxic growth environments, and growth media that more closely resemble the tumor microenvironment. In vivo models may also be improved upon by using induced models, rather than xenograft models, as well as by using orthotopic xenografts to better model disease development in more relevant biological contexts. Indeed, recent studies in our lab and others suggest that exogenous growth factors and nutrients that might be available in the tumor microenvironment may protect cancer cells from cell death and support their growth in the context of mitochondrial Ca^2+^ signaling blockade (Fernandez Garcia et al. 2023; Wang et al. 2022; Weissenrieder et al. 2025). Support of tumor growth by stromal and immune cells has become an area of intense research over recent years, particularly in highly stromal malignancies like pancreatic cancer (Li et al. 2018; Zhao et al. 2023b). Differences in metabolite or growth factor availability may alter sensitivity and resistance to pathway alterations in unexpected ways. For example, we have shown that exogenous TGFβ, which is secreted by both tumor cells and cancer‐associated fibroblasts, protects against growth defects in MCU‐knockout pancreatic cancer cells (Weissenrieder et al. 2025). This suggests that MCU inhibition may not be an appropriate monotherapy target in pancreatic cancer, though co‐inhibition of MCU and TGFβ signaling could be considered. Modulation of mitochondrial Ca^2+^ homeostasis in tandem with traditional chemotherapeutics, antimetabolic therapy, or growth factor inhibition should be explored as possible strategies for cancer‐selective synergistic (or additive) effects to reduce cell growth and metastasis with less opportunity for systemic toxicity. Such approaches would be consistent with recent trends in cancer treatment, which often rely on cocktails of multiple compounds targeting distinct mechanisms to inhibit multiple pathways at once to enhance treatment response and reduce the development of resistance, which often rapidly occurs in the context of monotherapy.

In summary, modulation of mitochondrial Ca^2+^ homeostasis may present a valuable target for therapeutic development in cancer. Cancer cell metabolism, energy availability, cell growth, and metastasis are all closely interrelated, with mitochondrial Ca^2+^ signaling providing key regulation of these processes. The importance of these relationships are difficult to overstate, with further research warranted. Improvements in small molecule development, inducible knockout models, cell culture conditions, and animal model generation will be critical for future work on a path to translation of therapeutic treatments for patients.

## Conflicts of Interest

JSW and JKF declare no conflicts of interest.

## Data Availability

Data sharing not applicable to this article as no datasets were generated or analyzed during the current study.
